# Exercise increases TCA intermediate concentrations during low‐calorie diet independent of insulin resistance among women with obesity

**DOI:** 10.14814/phy2.15987

**Published:** 2024-04-01

**Authors:** Tristan J. Ragland, Steven K. Malin

**Affiliations:** ^1^ Department of Health, Human Performance and Recreation Pittsburg State University Pittsburg Kansas USA; ^2^ Department of Kinesiology and Health Rutgers University New Brunswick New Jersey USA; ^3^ Department of Kinesiology University of Virginia Charlottesville Virginia USA; ^4^ Division of Endocrinology, Metabolism & Nutrition Rutgers University New Brunswick New Jersey USA; ^5^ New Jersey Institute for Food Nutrition and Health Rutgers University New Brunswick New Jersey USA; ^6^ Institute of Translational Medicine and Science Rutgers University New Brunswick New Jersey USA

**Keywords:** amino acids, diabetes, interval training, obesity, tricarboxylic acid cycle

## Abstract

Tricarboxylic acid cycle intermediates (TCAi) have been proposed to act as myokines that influence energy metabolism. We determined if 2‐weeks of low‐calorie diet with interval exercise (LCD + INT) would increase TCAi more than a low‐calorie diet (LCD). Twenty‐three women were randomized to 2‐weeks of LCD (*n* = 12, 48.4 ± 2.5 years, 37.8 ± 1.5 kg/m^2^, ~1200 kcal/d) or LCD + INT (*n* = 11, 47.6 ± 4.3 years, 37.9 ± 2.3 kg/m^2^; 60 min/d supervised INT of 3 min 90% & 50% HRpeak). TCAi and amino acids (AA) were measured at 0 min of a 75 g OGTT, while glucose, insulin, and FFA were obtained at 0, 30, 60, 90, 120, and 180 min to assess total area under the curve (tAUC_180min_) and insulin resistance (IR; tAUC_180min_ of Glucose × Insulin). Fuel use (indirect calorimetry) was also collected at 0, 60, 120, and 180 min as was fitness (VO_2_peak) and body composition (BodPod). Treatments reduced weight (*p* < 0.001), fasting RER (*p* = 0.01), and IR (*p* = 0.03), although LCD + INT increased VO_2_peak (*p* = 0.02) and maintained RER tAUC_180min_ (*p* = 0.05) versus LCD. Treatments increased FFA tAUC_180min_ (*p* = 0.005), cis‐aconitate, isocitrate, and succinate (*p* ≤ 0.02), as well as reduced phenylalanine and tryptophan, cysteine (*p* ≤ 0.005). However, LCD + INT increased malate, citrate, α‐ketoglutarate, and alanine more than LCD (*p* ≤ 0.04). Thus, INT enhanced LCD effects on some TCAi in women with obesity independent of IR.

## 1 INTRODUCTION

Decreased tricarboxylic acid intermediates (TCAi) have been reported in individuals with obesity and Type 2 diabetes (T2D) (Whytock et al., 2023; Hwang et al., 2010; Kulkarni et al., 2012; van de Weijer et al., 2013; Zou et al., 2019). Some (Menshikova et al., 2018), but not all (Whytock et al., 2023), have proposed that lower TCAi between healthy participants compared to those with obesity or T2D relate to mitochondrial dysfunction as well as insulin resistance. Although weight loss achieved by caloric restriction and/or exercise, lowers insulin resistance (Amati et al., 2009; Menshikova et al., 2005), and increases the action of insulin to suppress fatty acid oxidation to a similar degree in individuals with obesity (Corpeleijn et al., 2008), the role of caloric restriction on mitochondrial physiology is somewhat unclear. For instance, in healthy individuals reduced caloric intake improves mitochondrial biogenesis compared to overweight adults, although the same intervention did not increase citrate synthase, an indicator of TCA upregulation, in either group (Civitarese et al., 2007). In contrast, exercise is considered to promote mitochondrial function (Memme et al., 2021; Safdar et al., 2011; Silva et al., 2009). Together, these findings suggest diet and exercise may act through distinct mechanisms to alter mitochondrial physiology in relation to reductions in insulin resistance.

Most diet and/or exercise studies have focused on aspects of the mitochondria that relate to enzyme activity or cellular energetics, with less attention on TCAi per se. This is physiologically and clinically relevant since TCAi not only may reflect mitochondrial capacity to oxidize carbohydrate and fat, but also act as potential myokines (Maurer et al., 2021). Indeed, immunologic and cancer work have provided unique insight towards these TCAi as factors in metabolic programing of tumor cells (Choi et al., 2021a; Ryan et al., 2019). Given that exercise raises metabolic flux through the TCA cycle, it would not be surprising to consider that TCAi act as myokines to coordinate regulation of multiple tissues for reduction in insulin resistance as well as influence on fuel utilization. Indeed, Neilsen et al. reported that citrate, isocitrate, succinate, fumarate and malate all are released from skeletal muscle after exercise consisting of cycling and/or one‐legged leg extensions (Nielsen & Bloch Thomsen, 1979). Yet, despite an acute bout of exercise raising TCAi (Sahlin et al., 1990), training seems to decrease the TCAi response to a single exercise bout (Dawson et al., 2003; Howarth et al., 2004). While this later observation would align with improved fat oxidation capacity postexercise training, to date no exercise training studies prior to clinically meaningful weight loss have been compared to diet alone on circulating TCAi. Moreover, a low‐calorie diet (LCD) has been established to raise protein oxidation such that there is a rise in endogenous circulating amino acids (Collet et al., 2017). This rise in endogenous amino acids within the plasma may influence the TCAi pool, thereby influencing the circulating levels. Previously we reported that 2‐weeks of LCD raised fasting fat oxidation and lowered insulin resistance comparatively to an energy matched LCD + INT treatment arm in women with obesity (Gilbertson et al., 2019). Whether circulating amino acids relate with changes in plasma TCAi levels after LCD and/or LCD + INT remains unknown. Thus, we tested the hypothesis that LCD + INT would raise circulating TCAi concentrations compared to LCD alone, and this rise in TCAi would relate to changes in energy metabolism, insulin resistance, and amino acids.

## 2 METHODS

### 2.1 Participants

Twenty‐three females were randomized into LCD (*n* = 12, 48.4 ± 2.5 years, 37.8 ± 1.5 kg/m^2^) or LCD + INT (*n* = 11, 47.6 ± 4.3 years, 37.9 ± 2.3 kg/ m^2^). Some of these cardiometabolic data were previously reported (Eichner et al., 2019; Heiston et al., 2019; Malin et al., 2020; Ragland & Malin, 2023) but are shown here for interpreting the relevance of TCAi changes in association with lower insulin resistance. Those participants with adequate plasma samples for TCAi analysis were included in this analysis. Volunteers were recruited from the local community using social media and advertisements. Screenings consisted of a 12‐lead electrocardiogram (EKG) exercise stress test, medical history, physical examination, as well as blood chemistry analysis. Participants were excluded if they were weight unstable (>2 kg) for a least 3 months prior to enrollment, engaged in exercise (>60 min/week), pregnant, or had known cardiovascular disease, T2D, cancer, contraindications to exercise (e.g., musculoskeletal injuries), and/or taking medications (e.g., metformin, acarbose, GLP‐1 agonists, etc.) known to affect glucose homeostasis. All participants gave their written and verbal informed consent before study participation. Participants were also given a hard copy of the informed consent with their signature and that of a research member confirming explanation of the study. The study was conducted in accordance with the Declaration of Helsinki, and the protocol was approved by the University Ethics Committee (IRB‐HSR # 18316).

### 2.2 Cardiorespiratory fitness and body composition

A cycle ergometer with indirect calorimetry (CareFusion, V_max_ CART, Yorba Linda, CA, USA) was used to determine cardiorespiratory fitness (VO_2_peak) as described before by our laboratory (Howe et al., 2014). Workload was increased by about 25 watts every 2 min until the participant met volitional exhaustion, respiratory exchange ratio >1.1 or cadence <60 rpm. Heart rate (HR) and blood pressure were obtained throughout the test. Women were instructed to fast for approximately 4 h prior to body composition assessment. A digital scale was first used to measure body weight to the nearest 0.01 kg. Height was then measured with a stadiometer. Body fat and fat‐free mass (FFM) was determined using the BodPod (BodPod, Cosmed, CA, USA).

### 2.3 Metabolic control

Participants were instructed to refrain from caffeine and alcohol, as well as strenuous exercise 48 h prior to clinical testing. Participants were also asked to abstain from taking any medications or dietary supplements 24 h prior to reporting to the Clinical Research Unit. The last training bout was performed approximately 24 h before post‐intervention metabolic testing.

### 2.4 Oral glucose tolerance test

Participants arrived at the Clinical Research Unit after an overnight fast and underwent a 180 min 75 g oral glucose tolerance test (OGTT) (Fisher Scientific®, Waltham, MA, USA, Catalog # 40–122‐3FB). Blood samples for TCAi and amino acids were obtained from an antecubital vein at baseline. Additional blood draws occurred at 0, 30, 60, 90, 120, and 180 min for glucose, insulin, and free fatty acids (FFA). Insulin resistance (IR) was calculated by multiplying glucose by insulin total area under the curve (tAUC_180min_), which was subsequently reported as 10^6^ for ease of interpretation (Malin et al., 2013). tAUC_180min_ was calculated via the trapezoidal method. Respiratory exchange ratio (RER) was also collected using indirect calorimetry at 0, 60, 120, and 180 min of the OGTT to estimate fuel utilization.

### 2.5 Low‐calorie diet

Details of the protocol have been previously reported (Heiston et al., 2019; Malin et al., 2020; Ragland & Malin, 2023). But in short, 3 days prior to the LCD intervention, participants were given a food‐log to categorize their habitual diet. For the duration of the 13‐day intervention, participants were provided with meal replacement shakes for breakfast and lunch (Ensure® Abbott Laboratories, Lake Bluff, IL, USA, 8 fl. Oz procured via Amazon Marketplace®); providing 160 kcal, 16 g protein, 2 g fat, 19 g carbohydrate, two 100 kcal snack options per day, and participants were instructed to consume a sensible meal (e.g., lean protein with vegetables or salad) consisting of less than 600 kcal for dinner each day. During the intervention, participants were asked to keep a detailed food log and were instructed on the proper means of cataloging their food. All the empty meal replacement containers, along with the 13‐day food records were returned post intervention to assess compliance. The difference between the average 3‐day pre‐intervention food logs and the 13‐day average food intake was used to calculate the caloric deficit.

### 2.6 Low‐calorie diet and interval exercise

Women randomized to LCD + INT performed 12 sessions of INT exercise over the 13‐day invention, with one rest day around day seven. All exercise sessions were supervised and consisted of cycling for 3 min intervals of 50% and 90% of heart rate peak (HRpeak) for a total of 60 min. Workload was determined based on the participant's HR and adjusted accordingly to meet and maintain the desired intensity. After each exercise session, a mixed‐meal shake (Ensure®Abbott Laboratories, Lake Forest, IL, USA, 8 fl. oz; providing 350 kcal, 13 g protein, 11 g fat, 50 g carbohydrate) was provided to offset exercise energy expenditure and match the energy deficit observed in LCD alone as described before (Malin et al., 2020; Ragland & Malin, 2023).

### 2.7 Biochemical analysis

Blood glucose was collected in lithium‐heparinized vacutainers and immediately analyzed by a glucose oxidase assay (YSI Instruments 2700, Yellow Springs, OH, USA, SKU # 525000). Insulin was collected with aprotinin and analyzed via ELISA (Millipore, Billerica, MA Catalog # EZHI‐14K). FFA were collected in EDTA and analyzed via colorimetric assay (FujiFilm, Lexington, MA Catalog # 999‐34691, 995‐34791, 991‐34891, 993‐35191). TCAi and amino acids were determined from blood collected in EDTA vacutainers. Plasma samples (500 μL) were immediately centrifuged for 10 min at 15,000 g 4°C and stored at −80°C for later analyses. TCAi and amino acids were assessed as described previously (Remchak et al., 2022). Briefly, TCAi were determined via targeted metabolomics performed by the Mayo Clinic Metabolomics Core Facility via gas chromatography/mass spectrometry on an Agilent 5975C GC/MS. However, oxaloacetic acid is not reported due to technical difficulties (i.e., high signal to noise ratio). Likewise, amino acids were analyzed by flow injection tandem mass spectrometry. Concentrations of amino acids as well as fumaric acid (m/z 287.1), succinic acid (m/z 289.1), oxaloacetic acid (m/z 346.2), ketoglutaric acid (m/z 360.2), malic acid (m/z 419.3), cis‐aconitic acid (m/z 459.3), and citric acid (m/z 591.4), isocitric acid (m/z 591.4) were measured against a 7‐point calibration curve that underwent the same derivatization (Remchak et al., 2022).

### 2.8 Statistical analysis

Analysis was performed using SPSS version 27 (IBM 26th Edition). Baseline data were analyzed with independent two‐tailed t‐tests to determine possible differences between groups at baseline. If baseline differences were observed, it served as a co‐variate to confirm treatment effects. A two‐way repeated measures ANOVA was used to determine both treatment effect as well as group by treatment effect. Pairwise comparison was performed if a group × time interaction was observed using two‐tailed paired or independent t‐tests to assess within treatment effects or the delta (post‐pre) of the treatment when appropriate. Effect sizes were also calculated via eta squared (*η*2) to assess physiologic relevance and interpreted as small = 0.01, medium = 0.06, and large = 0.14. Data are expressed as mean ± SD. Significance was accepted as *p* ≤ 0.05.

## 3 RESULTS

### 3.1 Participant characteristics

LCD and LCD + INT reduced caloric intake during the intervention as reported previously (Ragland & Malin, 2023). Both treatments reduced weight (time effect; *p* < 0.001, *η* ^2^ = 0.70) and body fat (time effect; *p* < 0.001, *η* ^2^ = 0.49), although there was no difference in age (*p* = 0.87, *d* = 0.06), FFM (*p* = 0.20, *η* ^2^ = 0.07) or waist circumference (*p* = 0.96, *η* ^2^ = 0.00). Fitness increased following LCD + INT only (interaction effect; *p* = 0.03, *η* ^2^ = 0.21) and this was confirmed with pairwise analysis with a slight decrease after LCD compared to an increase following LCD + INT (Table 1). Exercise session compliance was approximately 99.3% for the LCD + INT groups, as reported previously (Gilbertson et al., 2019).

**TABLE 1:** Anthropometrics and Fitness Following LCD versus LCD + INT.

	LCD		LCD ± INT		T	G × T
	Pre	Post	Pre	Post		
N	12	‐	11	‐		
Age (years)	48.4 ± 9.0	‐	47.6 ± 14.2	‐		
Weight (kg)	101.8 ± 17.3	99.5 ± 16.9	104.6 ± 22.8	103.1 ± 22.8	<0.001	0.10
Body Mass Index (kg/m^2^)	37.8 ± 5.1	37.0 ± 5.1	37.9 ± 7.6	37.4 ± 7.6	<0.001	0.10
Waist Circumference (cm)	114.8 ± 12.4	115.6 ± 12.4	111.9 ± 18.2	111.0 ± 16.9	0.96	0.06
Body Fat (%)	51.1 ± 4.5	50.2 ± 5.1	49.0 ± 8.2	48.1 ± 8.6	<0.001	0.88
Fat Free Mass (kg)	48.9 ± 4.5	48.5 ± 4.1	51.9 ± 9.2	51.9 ± 8.9	0.20	0.42
VO_2_peak (L/min)	1.8 ± 0.3	1.6 ± 0.3	1.8 ± 0.3	2.0 ± 0.3^a^	0.72	0.01
VO_2_peak (mL/kg/min)	18.1 ± 3.4	17.5 ± 3.8	18.7 ± 5.3	20.1 ± 5.6^a^	0.34	0.02
VO_2_peak (mL/FFM‐kg/min)	36.8 ± 4.5	33.7 ± 5.8	36.4 ± 6.3	38.6 ± 5.9^a^	0.69	0.01

*Note*: Data expressed as mean ± SD. VO_2_peak: highest measured oxygen uptake. No statistical difference in age between groups, *p* = 0.87. T, *p*‐value for time effect; G × T, *p*‐value for group × time effect.

^a^ Delta (Post‐pre) compared between groups (*p* < 0.05).

### 3.2 Glucose metabolism and fuel use

Both treatments decreased fasting glucose (time effect; *p* = 0.03, *η* ^2^ = 0.19) and there was no effect on glucose tolerance (time effect; *p* = 0.91, *η* ^2^ = 0.001). Fasting insulin was unchanged after the interventions (time effect; *p* = 0.18, *η* ^2^ = 0.08), however, both treatments reduced insulin tAUC_180min_ (time effect; *p* = 0.005, *η* ^2^ = 0.36). Likewise, insulin resistance was reduced following both LCD and LCD + INT (time effect; *p* = 0.03, *η* ^2^ = 0.22). Fasting FFAs were not influenced by either treatment (*p* = 0.17), but postprandial levels were higher as reflected by tAUC_180min_ (time effect; *p* = 0.005, *η* ^2^ = 0.33). Fasting RER was reduced by LCD and LCD + INT (time effect; *p* = 0.04, *η* ^2^ = 0.19) as well as tAUC_180min_ (time effect; *p* < 0.001, *η* ^2^ = 0.61), although RER was maintained more after LCD + INT than LCD (interaction effect; *p* = 0.05, *η* ^2^ = 0.18). Pairwise analysis showed that LCD alone experienced a significant reduction (*p* = 0.002) while LCD + INT maintained RER (*p* = 0.07, Table 2).

**TABLE 2:** Glucose regulation and fuel utilization following LCD and LCD + INT.

	LCD		LCD ± INT		T	G × T
	Pre	Post	Pre	Post		
N	12	11	‐	‐
Fasting						
Glucose (mg/dL)	97.0 ± 5.2	94.2 ± 8.2	96.9 ± 8.6	92.9 ± 7.1	0.03	0.70
Insulin (μU/mL)	15.3 ± 7.2	22.3 ± 20.4	14.3 ± 10.6	18.1 ± 12.6	0.18	0.40
FFA (mEq/L)	0.4 ± 0.1	0.5 ± 0.1	0.5 ± 0.1	0.6 ± 0.1	0.17	0.31
RER (a.u.)	0.84 ± 0.0^a^	0.80 ± 0.0	0.79 ± 0.0	0.77 ± 0.0	0.01	0.30
Postprandial						
Glucose tAUC_180min_ (mg/dL)	20482.5 ± 3345.6	20550.7 ± 4303.7	22612.2 ± 3446.9	22416.6 ± 4128.8	0.91	0.81
Insulin tAUC_180min_ (μU/mL)	14055.6 ± 6085.4	12414.8 ± 6001.6	17078.4 ± 5688.4	12905.4 ± 2951.0	0.005	0.17
Insulin Resistance	229.3 ± 146.1	265.4 ± 157.6	375.8 ± 157.8	289.8 ± 131.3	0.03	0.26
FFA tAUC_180min_ (mEq/L)	30.7 ± 8.6	38.6 ± 13.8	32.6 ± 14.2	40.8 ± 12.5	0.005	0.94
RER tAUC_180min_ (a.u.)	162.2 ± 5.1	149.87 ± 8.6*	156.2 ± 9.2	149.2 ± 6.9^b^	<0.001	***0.05***

*Note*: Data expressed as mean ± SD. T, *p*‐value for time effect; G × T, *p*‐value for group × time effect.

^a^ Significant difference between groups at baseline, hence co‐varied for ANOVA.

^b^ Delta (Post‐pre) compared between groups (*p* < 0.05).

* Compared with Pre‐test *p* < 0.05.

### 3.3 TCAi

**FIGURE 1:**
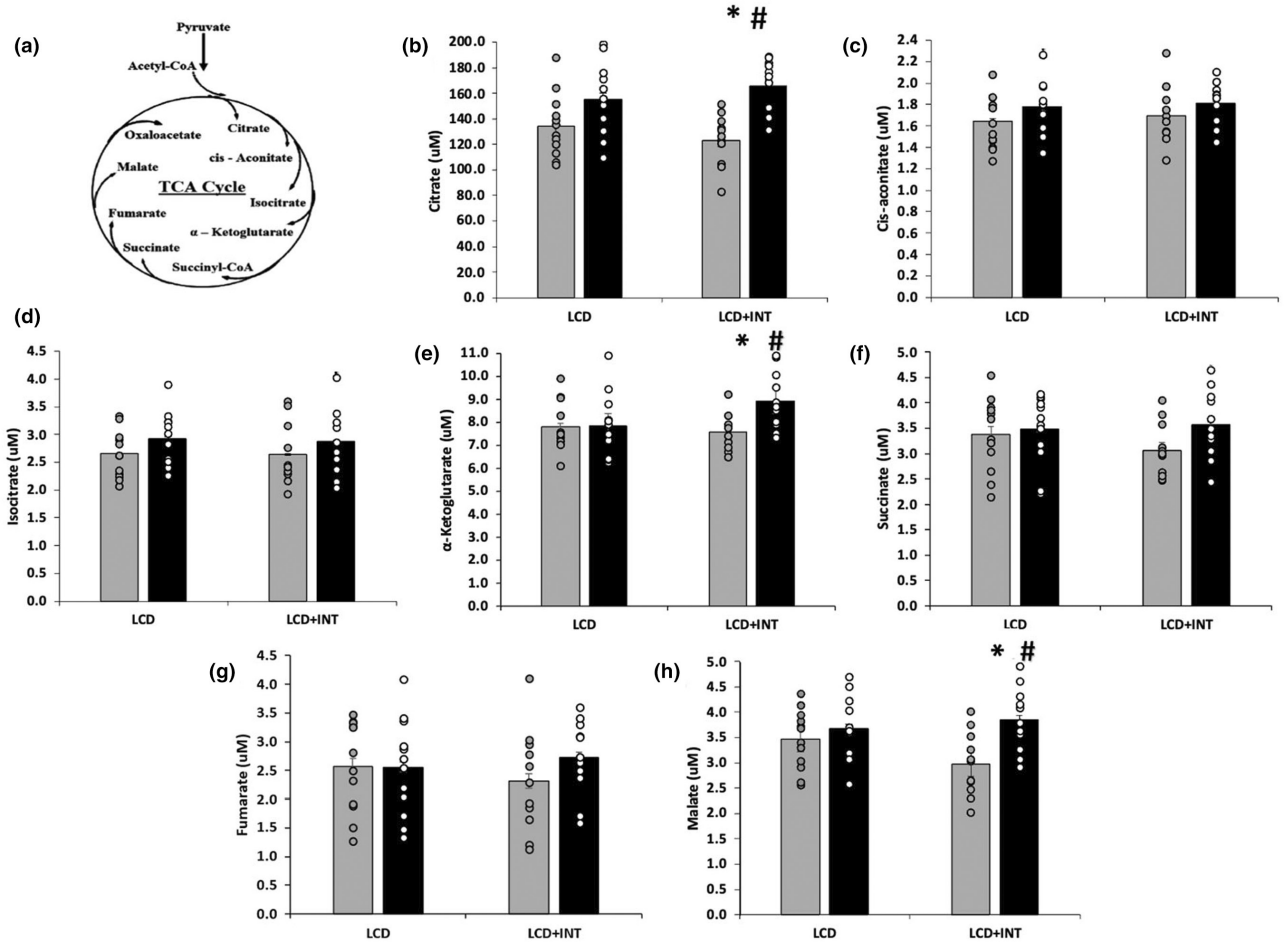
Data expressed as mean and circles denote individual data points. (a) TCA cycle, (b) citrate, (c) cis‐aconitate, (d) isocitrate, (e) α‐ketoglutarate, (f) succinate, (g) fumarate, (h) malate. Note that citrate, cis‐aconitate, isocitrate, succinate and malate were found to have main effects of time, *p* < 0.05. ^#^Group × time effect *p* < 0.05. *Compared to Pre‐test, *p* < 0.05.

Both LCD and LCD + INT increased cis‐aconitate (time effect; *p* = 0.02, *η* ^2^ = 0.25), isocitrate (time effect; *p* = 0.02, *η* ^2^ = 0.22), and succinate (time effect; *p* = 0.01, *η* ^2^ = 0.25) after the intervention. However, LCD + INT increased α‐ketoglutarate more than LCD (*p* = 0.009, *η* ^2^ = 0.30). Pairwise analysis indicated that LCD + INT alone increased α‐ketoglutarate (*p* = 0.009) compared with no change after LCD (*p* = 0.95). Furthermore, although both LCD and LCD + INT increased citrate (time effect; *p* < 0.001, *η* ^2^ = 0.65, Figure 1), LCD + INT increased citrate more than LCD (interaction effect; *p* = 0.04, *η* ^2^ = 0.17). This was confirmed with pairwise analysis showing that LCD + INT alone increased citrate (*p* < 0.001) while LCD did not (*p* = 0.06). Likewise, both groups raised malate (time effect; *p* < 0.001, *η* ^2^ = 0.41) but LCD + INT increased malate more than LCD (interaction effect; *p* = 0.02, *η* ^2^ = 0.20). Pairwise analysis revealed that LCD + INT alone increased malate (*p* = 0.004) and LCD did not (*p* = 0.40).

### 3.4 AA concentrations

AAs were organized by essential and nonessential AAs (Table 3). Only the essential amino acids phenylalanine (time effect; *p* = 0.004, *η* ^2^ = 0.33) and tryptophan (time effect; *p* = 0.005, *η* ^2^ = 0.32) were reduced following LCD and LCD + INT. Further, both treatments decreased the nonessential amino acid, cysteine (time effect; *p* = 0.005, *η* ^2^ = 0.31), and LCD decreased while LCD + INT increased alanine (interaction effect; *p* = 0.03, *η* ^2^ = 0.20). Pairwise analysis specified that LCD alone decreased (*p* = 0.003) alanine while LCD + INT maintained (*p* = 0.55) alanine concentrations.

**TABLE 3:** Amino acids following LCD versus LCD + INT.

	LCD		LCD ± INT		T	G × T
	Pre	Post	Pre	Post		
Essential Amino Acids						
Histidine (μM)	65.6 ± 24.8	62.1 ± 21.2	70.9 ± 23.2	72.7 ± 17.2	0.79	0.42
Isoleucine (μM)	54.1 ± 6.9	55.8 ± 8.6	50.7 ± 9.9	53.5 ± 10.2	0.13	0.70
Leucine (μM)	103.1 ± 13.2	101.7 ± 11.6	99.6 ± 15.2	100.5 ± 17.2	0.90	0.59
Lysine (μM)	157.5 ± 21.8	161.7 ± 15.5	157.2 ± 18.2	171.2 ± 24.5	0.06	0.30
Methionine (μM)	20.5 ± 1.9	19.8 ± 1.9	19.85 ± 0.9	20.2 ± 2.6	0.76	0.28
Phenylalanine (μM)	51.9 ± 6.3	49.3 ± 5.9	52.3 ± 5.6	50.5 ± 6.3	0.004	0.54
Threonine (μM)	134.1 ± 19.8	141.2 ± 27.5	117.4 ± 19.8	113.2 ± 26.5	0.75	0.24
Tryptophan (μM)	51.7 ± 3.9	47.9 ± 3.6	48.9 ± 8.9	46.3 ± 10.6	0.005	0.56
Valine (μM)	204.1 ± 33.8	205.4 ± 28.5	196.5 ± 34.1	198.6 ± 24.5	0.69	0.92
Nonessential Amino Acids						
Alanine (μM)	360.8 ± 54.7^a^	297.4 ± 32.5*	302.2 ± 60.3	316.9 ± 53.0^b^	0.001	0.03
Arginine (μM)	76.1 ± 14.2	73.2 ± 14.5	76.0 ± 15.9	81.1 ± 17.2	0.73	0.21
Asparagine (μM)	67.1 ± 10.9	68.0 ± 11.2	65.6 ± 12.2	70.7 ± 16.2	0.21	0.37
Aspartic Acid (μM)	1.3 ± 1.5	1.2 ± 0.2	1.0 ± 0.3	1.0 ± 0.2	0.50	0.53
Cysteine (μM)	20.5 ± 5.3	16.3 ± 3.9	16.7 ± 2.9	15.3 ± 3.3	0.005	0.15
Glutamic Acid (μM)	38.1 ± 13.5	35.7 ± 11.2	38.4 ± 19.2	35.3 ± 18.5	0.44	0.92
Glutamine (μM)	464.5 ± 68.3	462.8 ± 70.6	446.0 ± 67.3	464.9 ± 59.0	0.52	0.45
Glycine (μM)	264.9 ± 103.1	258.6 ± 114.7	186.2 ± 62.3	194.4 ± 55.3	0.90	0.34
Proline (μM)	188.2 ± 56.7^a^	178.6 ± 63.0	167.11 ± 52.4	153.7 ± 40.4	0.22	0.58
Serine (μM)	93.8 ± 15.5	99.1 ± 20.8	80.1 ± 11.9	84.1 ± 15.9	0.09	0.81
Tyrosine (μM)	58.9 ± 7.2	57.4 ± 7.2	60.6 ± 12.2	59.2 ± 9.9	0.46	0.99

*Note*: Data expressed as mean ± SD. T, *p*‐value for time effect; G × T, *p*‐value for group × time effect.

^a^ Significant difference between groups at baseline, hence co‐varied for ANOVA.

^b^ Delta (Post‐pre) compared between groups (*p* < 0.05).

* Compared with Pre‐test *p* < 0.05.

### 3.5 Correlations

Neither TCAi and/or AA were significantly related to weight loss, fitness or insulin resistance changes following LCD and LCD + INT (data not shown). However, lower RER tAUC_180min_ related to higher cis‐aconitate (*r* = −0.43, *p* = 0.04). Elevated FFA tAUC_180min_ also related to higher succinate (*r* = 0.44, *p* = 0.03) and citrate (*r* = 0.43, *p* = 0.04).

## 4 DISCUSSION

Herein, we show that LCD and LCD + INT increased plasma cis‐aconitate, isocitrate, and succinate after treatment. Interestingly, while both treatments raised citrate and malate, LCD + INT raised citrate, malate, and α‐ketoglutarate more than LCD only. Interestingly, LCD + INT maintain alanine compared with LCD that could favor these TCAi via anaplerosis. However, both treatments lowered the AAs phenylalanine, tryptophan, and cysteine comparably. While alanine was maintained after LCD + INT, alanine could contribute to elevations in pyruvate, which would feed forward to support citrate elevations. Together, these data suggest that interval exercise differentially influences some TCAi compared to LCD only. Consistent with this later idea, caloric restriction of 25% total daily energy needs, for 6 months, increases genes linked to mitochondrial biogenesis and function in people with excess body weight, but does not necessarily shift TCAi (citrate synthase), fat oxidation (beta‐hydroxyacyl‐CoA dehydrogenase), or electron transport chain (cyto‐chrome C oxidase II) enzymes in all studies (Civitarese et al., 2007; Menshikova et al., 2018). Nevertheless, exercise training raises mitochondria content, electron chain transport, and fatty acid oxidation (Menshikova et al., 2018), indicating potential alternative mechanisms by which exercise versus diet alter muscle metabolism. Exercise has been shown, in recent years, to increase several circulating TCAi above resting levels (Bowtell et al., 2007; Gibala et al., 1997, 1998; Zhang et al., 2019) in animals and healthy adults. In fact, citrate, succinate, fumarate, and malate have been reported to rise from muscle contraction during and/or immediately postexercise through use of arterio‐venous difference technique, and it has been shown that citrate (Hargreaves et al., 1991; Nielsen & Bloch Thomsen, 1979), succinate (Lewis et al., 2010; Reddy et al., 2020), and malate (Hu et al., 2020; Lewis et al., 2010) are readily released from the exercising muscle and cleared in the liver (Hu et al., 2020). Importantly, in the current work, the last exercise bout of training was performed 24 h prior to TCAi measurement. These data suggest rises in certain TCAi may relate more to repeated exercise sessions than, per se, the residual last bout. In either case, an increased flux of TCAi including isocitrate, cis‐aconitate, and alpha‐ketoglutarate has been suggested to be important for physiologic regulation of tissues (Maurer et al., 2021). Indeed, TCAi released from the skeletal muscle can act in a paracrine/endocrine manner to enhance fuel utilization, insulin sensitivity, and mitochondrial content (Maurer et al., 2021). For instance, succinate has been shown to act on skeletal muscle to activate calcium signaling to enhance mitochondrial content and induce a fast‐ to slow‐twitch fiber characterization (Maurer et al., 2021; Reddy et al., 2020; Wang et al., 2019). In the current study, we show that succinate was increased following both LCD and LCD + INT, which was consistent with observations that fasting RER was comparably reduced (i.e., increased fat oxidation). On the contrary, citrate has been indicated to play a role immune regulation (Choi et al., 2021b; Ciccarone et al., 2017; Ryan et al., 2019) and influence the expression of inflammatory cytokines (Infantino et al., 2019). Collectively, these data suggest further investigation into the role of TCAi on cardiometabolic is warranted.

It is of interest to understand how LCD and LCD + INT influenced TCAi. Caloric restriction and exercise both create an energy deficit, thereby shifting reliance toward resting fat oxidation and increasing flux through the TCA cycle. It would thus be reasonable to anticipate weight loss to contribute to elevations in TCAi in the circulation. In the current study, we observed about 2% weight loss following the 2‐week intervention. This observation would be consistent with the notion that energy deficit may have promoted greater flux through the TCA cycle thereby increasing the TCA pool size and shifting balance of anaplerosis toward cataplerosis (Owen et al., 2002; Ryan et al., 2019). However, this seems unlikely to account entirely for the differential TCAi response since LCD + INT raised citrate, malate, and α‐ketoglutarate more than LCD alone. In contrast, aerobic fitness is coupled to mitochondrial respiration, and greater gains in VO_2_peak would potentially enhance TCAi elevations. Despite an approximate 7% rise in VO_2_peak with LCD + INT and no change with LCD, we observed no correlations with any circulating TCAi after the intervention. Certainly, association does not equal causation and the use of arterio‐venous balance techniques or muscle biopsies would provide more insight towards definitively concluding whether gains in aerobic fitness explain the differential TCAi within our study.

Another possible explanation for why LCD + INT differentially increased citrate, malate, and α‐ketoglutarate relates to energetic stress on the mitochondria. Both groups underwent energy deficit, but only exercise elevated energy expenditure. While both LCD and LCD + INT lowered fasting RER like other studies (Menshikova et al., 2005, 2018; Remchak et al., 2022), it is worth noting that LCD + INT maintained postprandial RER more post‐intervention compared with LCD. This suggests that LCD + INT preserved postprandial carbohydrate oxidation and energy expenditure compared with LCD. It worth noting that we previously measured plasma lactate during the OGTT following LCD and LCD + INT and reported that lactate levels were comparably lowered (Gilbertson et al., 2019). This is potentially relevant since lactate could contribute to formation of pyruvate for citrate conversion. Without using lactate stable isotopes, we are unable to determine if the decrease in lactate following LCD is due to less CHO oxidation while the reduction of lactate after LCD + INT is due to greater formation of pyruvate/citrate. As such, this preservation in energy expenditure during at least the postprandial state could relate to elevating TCAi levels after LCD + INT because a reduction in energy expenditure seen following caloric restriction might be anticipated to lower TCAi levels. While we did not previously detect decreases in resting metabolic rate using following LCD or LCD + INT (Malin et al., 2020), it is accepted that fat yields less energy when scaled per unit of oxygen compared to carbohydrate (i.e., about 4.7 kcal vs. 5.0 per 1 L of O_2_). Thus, it is possible that those after LCD had subtle reductions in energy expenditure compared with preservation in metabolic rates following LCD + INT and this influenced TCAi levels seen here. In either case, our collective work suggests that LCD + INT may differentially support insulin action to oxidize glucose compared with LCD. In particular, these data highlight that LCD likely stored glucose to a greater relative extent than LCD + INT. Interestingly, lower RER tAUC_180min_ and higher FFA tAUC_180min_ each were associated with cis‐aconitate, succinate and citrate, respectively. This may imply that FFA rose in response to energy deficit and supported to some extent oxidative energy expenditure during the OGTT to enable glucose to be taken up for non‐oxidative metabolism. In contrast, the greater carbohydrate oxidation seen after LCD + INT may have raised metabolic flux through the TCA cycle and promoted elevated release of some TCAi. Physiologically, this is reasonable given beta‐oxidation is linked to Complex II of the mitochondria (Memme et al., 2021; Silva et al., 2009), thereby aligning succinate and citrate metabolism as the first TCAi after acetyl‐CoA is combined with oxalacetate (Maurer et al., 2021). Subsequently, further work is required to understand how these TCAi may act to coordinate insulin‐mediated glucose uptake and/or metabolism.

It is important to consider the potential limitations of the current work. This investigation was a secondary analysis in women with obesity. We accept potential for Type 1 error rates at a *p* < 0.05, and the conclusions cannot be generalized to men with obesity nor to individuals with overt disease (e.g., T2D, hypertension, etc.). Also, TCAi in the current investigation were collected in the fasted state prior to an OGTT. Therefore, we are unable to delineate what effect glucose ingestion may have on circulating TCAi after training. However, prior work indicated that TCAi were not altered from the basal state following insulin stimulation in myotubes derived from individuals with obesity (Zou et al., 2019). Thus, it would seem unlikely that TCAi in circulation changed after the glucose ingestion. Although exercise increases release of TCAi from contracting skeletal muscle into circulation, we cannot rule out the potential for other tissues (e.g., liver, adipose, etc.) releasing or clearing TCAi. Also, this investigation utilized interval training on a cycle ergometer, and it is unclear if similar outcomes would result from continuous endurance training or resistance training. We used the OGTT to estimate insulin resistance and relying on glucose and insulin to estimate insulin resistance can be challenging. Both treatments herein lowered insulin tAUC_180min_ yet had no effect on glucose tAUC_180min_ averages. Variability though in glucose responses after LCD versus LCD + INT may explain underestimated changes in insulin resistance compared with use of the euglycemic clamp. Further, fuel utilization was assessed by indirect calorimetry versus stable isotopes. Lastly, this was a 2‐week intervention, and it is unclear if a longer‐term intervention of caloric restriction, with or without exercise, would have similar effects on circulating TCAi concentrations. Nevertheless, a strength of the current work is attempting to equate energy availability between LCD and LCD + INT, thereby isolating the effect of LCD + INT versus LCD on respective outcomes.

In conclusion, INT enhanced the effect of LCD on some circulating TCAi in women with obesity independent of reductions in insulin resistance. Thus, these findings indicate a possible differential shift in TCAi‐mediated signaling when exercise is combined with caloric restriction compared to a low‐calorie diet alone. Thus, further work examining how exercise vs. diet mediate improvements in insulin resistance and cardiometabolic health is warranted to optimize risk reduction for Type 2 diabetes and cardiovascular disease.

## FUNDING INFORMATION

Supported by NIH RO1‐HL130296, University of Virginia Thelma R. Swortzel Award and Diabetes Action Research and Education Award (SKM).

## CONFLICT OF INTEREST STATEMENT

The authors have no conflict of interest to declare.

## Data Availability

Corresponding author will provide data upon reasonable request.
